# Tumor-reactive T cells are licensed by dendritic cells located in spatially different tissues: implications for dendritic cell vaccines

**DOI:** 10.18632/oncotarget.27972

**Published:** 2021-08-03

**Authors:** Nadine Santana-Magal, Leen Farhat-Younis, Yaron Carmi

**Keywords:** dendritic cells, antigen presentation, T cells

Since the discovery of dendritic cells (DC), exploiting their unique ability to activate CD4^+^ and CD8^+^ T cells against cancer cells has held great promise [1, 2]. Nonetheless, despite countless attempts [3], DC-based vaccines have not yet lived up to expectations [4], beyond preventing tumor growth in prophylactic or adjuvant tumor settings [5, 6] and anecdotal success [7]. It remains unclear why the activation of DC, which are so prominent in activating tumor-reactive T cells, does not lead to eradication of established solid tumors.

The general wisdom suggests that DC take up tumor antigens at the tumor site and then migrate into the lymph nodes, where they present them to T cell. Since most tumors lack sufficient danger signals that allow DC to present tumor antigens in a stimulatory context, it is not surprising that the corresponding T cells are often skewed towards regulatory, or anergic phenotype. Consistent with that notion, most DC vaccines are based on stimulating DC *ex vivo*, followed by their infusion back to the patient. Traditional efforts focused on finding the most potent stimuli to mature and activate DC and on identifying the ideal unique, high-avidity tumor antigens that can be loaded on the DC [4, 8]. More recently, major efforts have been targeted at locating the most potent DC subset to stimulate reactive T cells [9]. DC are comprised of heterogeneous cell populations that differ from each other in their pattern-recognition receptors, tissue distribution, migratory patterns, and antigen-presentation capabilities [10, 11]. DC subsets such CD103^+^/CD141^+^ or CD8^+^ cDC are superior antigen-presenting cells, whose prevalence in tumors and blood is limited [12–14]. In contrast, MoDC are much more abundant and can be easily matured *ex vivo* from circulating monocytes [15]. While such efforts improve the prevalence of anti-tumor T cells in the circulation, eradication of established tumors remains out of reach. One possibility is that tumor-reactive T-cell clones are induced by DC yet are inhibited by the suppressive tumor microenvironment, or by intrinsic changes in tumor cells [16, 17].

In a recent paper, Santana-Magal and colleagues studied why once melanoma tumors exceed a certain size in mice, immunotherapies fail to eradicate them, despite the presence of tumor-reactive T cells in the periphery [18]. Using the photoactivatable Denrdra2 mice, which enable to fluorescently-label cells specifically in the tumors using optical fiber, she found that in contrast to the current paradigm, MoDC do not migrate from the tumor lesion to the draining lymph nodes. While migration of migratory and epidermal DC to the DLN is observed, it only occurs during early tumor onsets, and these cells are almost completely absent from late-stage tumors. At this stage, antigens predominantly reach the DLN trough passive drainage. Most importantly, Santana-Magal and colleagues’ work stresses that the main role of tumor-infiltrating DC is not to present tumor antigens, but rather to license the cytotoxic activity of infiltrating T cells.

**Figure 1:**
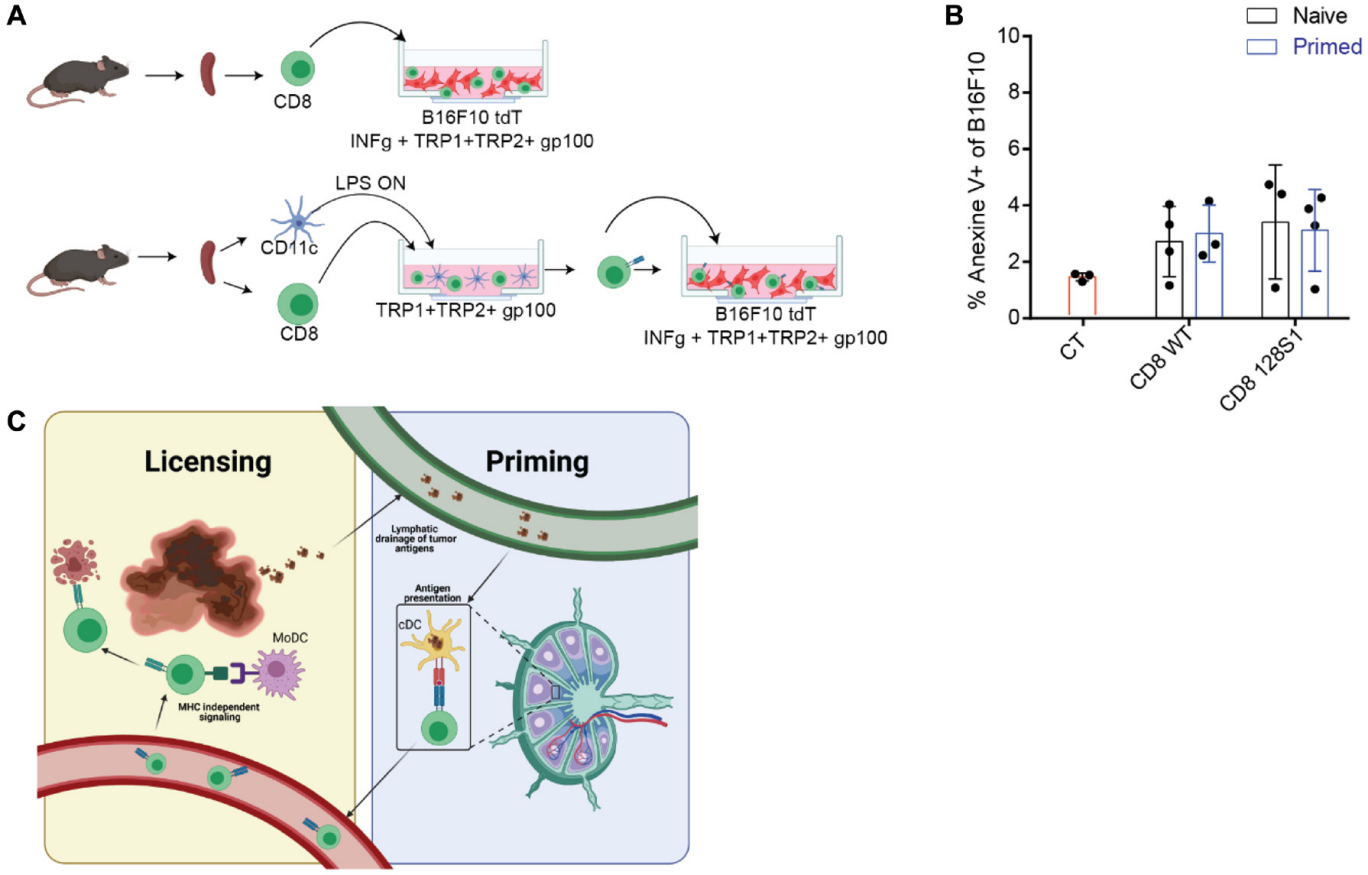
Activation of T cells by LN DC is not sufficient to elicit tumor cell lysis. (**A**) Experimental design- Splenic CD8^+^ T cells are isolated from naïve mice and incubated with IFNγ-stimulated tumor cells as such, or after incubation with activated LN-derived DC pulsed with tumor antigens. (**B**) Graph summarizes the percentages of B16F10 cells that express Annexin V following overnight incubation with splenic T cells. (**C**) Illustration of proposed T cell activation model. Illustrations were created with https://BioRender.com.

To further test that possibility, we isolated CD8^+^ T cells from control mice, tumor-vaccinated mice, or allogeneic mice and incubated them for three to four days with LPS-activated LN DC pulsed with a mixture of known melanoma antigens. Next, we isolated the T cells and incubated them with melanoma cells pre-activated with IFNγ and pulsed with the same MHC-I antigens used for priming (experimental design is illustrated in Figure 1A). Importantly, none of the primed T cells was capable of inducing melanoma cell killing (Figure 1B). However, the addition of MoDC to that co-culture induced a four-fivefold enhancement of the killing capacity of primed T cells, in an MHC-I independent manner (not shown). This simple experiment strongly supports the argument that a single signal, as potent as it may be, delivered by LN DC is not sufficient to overcome the intrinsic activation threshold to elicit the cytotoxic activity of T cells.

Over the past decade, a number of seminal studies demonstrated that TCR-MHC is more promiscuous than earlier thought, allowing each T cell to recognize several to hundreds of different peptides [19–21]. One potential consequence of these models is that licensing T cells to act in the lymph node will result in off-site cytotoxicity, or the lysing of antigen-presenting cells expressing similar antigens. The T-cell activation model suggested by Santana-Magal and colleagues (illustrated in Figure 1C) provides a different insight to the limited clinical benefit of DC vaccines and a simple explanation as to why LN DCs are not killed by their corresponding activated T cells. Consistent with previous publications emphasizing the spatial elements involved in induction of tumor immunity [15], we believe that potent DC vaccines must account for the need to stimulate the various DC subsets located at both the LN and tumor sites. One such approach was recently published by Ackerman and colleagues, utilizing tumor-binding antibodies coupled with a stimulatory agent. It may be that such a procedure, possibly in combination with the blocking of suppressor receptors on T cells, would elicit the full cytotoxic potential of infiltrating tumor-reactive T cells.
